# EOF and target PFAS analysis in surface waters affected by sewage treatment effluents in Berlin, Germany

**DOI:** 10.1007/s00216-022-04500-x

**Published:** 2023-01-12

**Authors:** Tengetile Nxumalo, Abdullah Akhdhar, Viktoria Mueller, Fabian Simon, Marcus von der Au, Antje Cossmer, Jens Pfeifer, Eva M. Krupp, Björn Meermann, Andrew Kindness, Jörg Feldmann

**Affiliations:** 1 Trace Element Speciation Laboratory Aberdeen (TESLA), Department of Chemistry, University of Aberdeen, Aberdeen, AB24 3UE UK; 2 Federal Institute for Materials Research and Testing, Division 1.1 – Inorganic Trace Analysis, Richard-Willstätter-Straße 11, 12489 Berlin, Germany; 3 Department of Chemistry, College of Science, University of Jeddah, P.O. 80327, Jeddah, 21589 Saudi Arabia; 4 Environmental Analytical Chemistry, Institute of Chemistry, TESLA, University of Graz, 8010 Graz, Austria; 5 The James Hutton Institute – Craigiebuckler, Aberdeen, AB15 8QH UK

**Keywords:** PFAS, HR-CS-GFMAS, ICP-MS, LC–MS/MS, EOF, Fluorine

## Abstract

Per- and polyfluoroalkyl substances (PFAS) are emerging organic pollutants and can occur in surface and groundwater. To identify the degree of pollution in surface water with PFAS, often targeted HPLC–ESI–MS/MS has been employed in which commonly 30–40 compounds are analyzed. However, other PFAS and organofluorines remain undetected. We sampled surface water of the river Spree and the Teltow Canal in Berlin, Germany, which are affected by the effluent discharge of wastewater treatment plants. Here, we employed high-resolution continuum source graphite furnace molecular absorption spectrometry (HR-CS-GFMAS) for measuring extractable organofluorines (EOF) and compared in a mass balance approach the total fluorine to the identified and quantified PFAS from the targeted analysis. The analysis highlights that the EOF are in the range expected for an urban river system (Winchell et al. in Sci Total Environ 774, 2021). However, downstream of an effluent discharge, the EOF increased by one order of magnitude, e.g., 40.3 to 574 ng F L^−1^, along the Teltow Canal. From our target analytes, mostly short-chained perfluorinated carboxylic acids and sulfonates occur in the water, which however makes up less than 10% of the EOF. The increase in EOF in the Teltow Canal correlates well with the increase of perfluorohexanoic acid (PFHxA), indicating that PFHxA is characteristic for the discharged EOF but not responsible for the increase. Hence, it points to PFHxA precursor discharge. The study highlights that EOF screening using HR-CS-GFMAS is necessary to identify the full scale of pollution with regard to PFAS and other organofluorines such as pharmaceutical compounds from the effluent of WWTPs.

**Supplementary Information:**

The online version contains supplementary material available at 10.1007/s00216-022-04500-x.

## Introduction

Per- and polyfluoroalkyl substances (PFAS) are a group of artificially made materials, manufactured since the 1950s and are widely used since then. Due to the strong C-F bond (485 kJ mol^−1^), the compounds have high thermal and chemical stability, highly resistant against degradation processes, making them bioaccumulative. In addition, these compounds have an amphiphilic nature, thus making them an excellent material for industrial and commercial appliances [2, 3]. Polyfluoroalkyl substances, often referred to as PFAS precursors, may undergo degradation processes resulting in perfluoroalkyl acids (PFAA), e.g., perfluorooctane sulfonic acid (PFOS) and perfluoro octanoic acid (PFOA), which are more stable in the environment [4–6]. Due to the increasing health concerns, PFOS is listed as a persistent organic pollutant (POP) [7]. The production of PFOS and PFOA and their precursor compounds was phased out [8]. Nevertheless, these compounds are still detected in the environment. As PFOA and PFOS are the most prevalent in the environment, their concentration is monitored in drinking water samples. The recommended limit by the Directive (EU) 2020/2184 of the European Parliament and of the Council of 16 December 2020 on the quality of water intended for human consumption is set to 0.10 µg L^−1^ and 0.50 µg L^−1^ for the sum of PFAS (sum of C_4_–C_13_ carboxylic and sulfonic acids) and total PFAS [9].

Since the first discovery of PFOS in animals by Giesy and Kannan [10], many kinds of PFAS and their precursors have been detected in drinking water [11], river water, fish [12, 13], vegetables [14], and even in humans [15]. Due to its bioaccumulative properties, they were voluntarily phased out from production [16], replacing long-chain (C > 8) PFAS to shorter chain length (C < 8), less bioaccumulative PFAS. However, these PFAS compounds are more hydrophilic and could be more mobile in the environment [17]. PFAS can often be found in the effluent water and biosolids of wastewater treatment plants (WWTPs) [18, 19]. There is evidence that WWTPs do not remove PFAS from wastewater efficiently [20]; hence, PFAS can enter the environment via wastewater effluent. In general, there is an increase in the concentration of perfluoroalkyl acids (PFAAs) observed in the effluent water from WWTPs that use biotreatment to destroy contaminants, as PFAS precursors can transform to PFAA during these biological processes, and can be considered as an important secondary PFAS source in the environment [18, 19, 21].

Due to their lack of chromophores, water solubility, low volatility, and excessive presence in consumables and instruments, PFAS measurement is challenging. Nonetheless, standard methods for analysis of water samples are available (e.g., US EPA 533, US EPA 8327, EPA 1633, ASTM D7979) [22–24]. The analysis is performed using high-performance liquid chromatography coupled to tandem mass spectrometry (HPLC–MS/MS); however with this approach, only those PFAS are analyzable that are easily ionisable and have standards available for (usually up to 30 analytes). This is a so-called targeted analysis approach. Total fluorine (TF) and extractable organofluorines (EOF) can be measured with different instruments, for example, combustion ion chromatography (CIC) [25–27] or high-resolution continuum source graphite furnace molecular absorption spectrometry (HR-CS-GFMAS) [28–32]. EOF measurement with both techniques provide comparable results; however, analysis with HR-CS-GFMAS was found to be less time-consuming and more sensitive [29]. For seawater samples from an uncontaminated site in Japan, EOF values determined with CIC of 93 ng F L^−1^ were found, whereas for a contaminated site, concentrations of 562 ng F L^−1^ were determined [25]. In surface water samples from Sweden from a fire training affected site, EOF concentrations determined with CIC reached up to 3930 ng F L^−1^. In the same study, industrial runoff samples were analyzed, and EOF concentrations of 1110 ng F L^−1^ were determined [33]. EOF concentrations determined using HR-CS-GFMAS were between 50 and 300 ng F L^−1^ in German rivers from Moselle and Rhine [31]. For surface water samples in the river Spree, Germany, EOF concentrations determined using HR-CS-GFMAS were between 50 and 550 ng F L^−1^ [29]. For another organofluorine sum parameter — the adsorbable organic fluorine (AOF) — concentrations of 880 to 1470 ng F L^−1^ were determined in surface water samples from different German rivers including Rhine, Main, and Danube [27]. In another study, AOF concentrations of surface water samples from seven rivers in Hesse, Germany, were between 2300 and 24,500 ng F L^−1^ [34]. AOF concentrations in the effluent of the WWTP in Germany were 2000 ng F L^−1^ [35].

A fluorine mass balance analysis pairs TF and EOF analyses with target analysis to assess the fractions of identifiable PFAS as well as the fractions of not-yet-identified or non-eluting PFAS. Many reports of PFAS in wastewater effluent and river water focuses on targeted analysis, which can underestimate the overall organofluorine concentrations [27]; thus, the EOF and the identifiable fractions remain unknown. The aim of this project is to study whether the use of HR-CS-GFMAS can assist to identify the extractable organofluorine pollution compared to targeted analysis for PFAS. Here as a case study, river and canal systems in Berlin, Germany, were sampled to demonstrate the importance of determining extractable organofluorines in addition to targeted PFAS analysis to identify whether effluents from waste water treatment plants are a significant source of PFAS and organofluorines in Berlin waterways.

## Material and methods

### Chemicals and materials

Deionized water with a resistivity of 18.2 MΩ cm from Merck (Darmstadt, Germany) was used for sample dilution and preparation of calibration solutions. Nitric acid (HNO_3_ 65%) was purchased from CHEM Solute (Rennigen, Germany), and HNO_3_ was diluted to 1.3% (v/v) for leaching and rinsing purposes. The fluoride standard for EOF analysis was prepared from 1 g L^−1^ sodium fluoride (NaF) water (Merck, Darmstadt, Germany). Methanol from Merck, Darmstadt, Germany, was used. Plastic tubes (15 mL, 50 mL) (VWR International, polypropylene centrifugal tubes) were used for standard and sample preparation. Both were leached with 1.3% (v/v) HNO_3_ solution for at least 12 h before using them. AAS sample vessels (polystyrene, 1.5 mL) were used from Analytik Jena (Jena, Germany). The samples were filtrated using 0.45-μm nitrocellulose from Lab Solute (Rennigen, Germany). Calcium ICP standard 1 g L^−1^ Ca(NO_3_)_2_ in HNO_3_ 2–3% from Merck (Darmstadt, Germany) was used for conditioning the graphite furnace. Gallium (III) nitrate hydrate (99.999% grade; Sigma-Aldrich, St. Louis, USA) was used as a forming-reagent at a concentration of 1 g L^−1^ Ga. Zirconium 1 g L^−1^ ICP standard NIST ZrOCl_2_ in HCl 7% was used for coating graphite furnace. Palladium (10 g L^−1^ (Pd (NO_3_)_2_/15% HNO_3_)) and magnesium 10 g L^−1^ (Mg (NO_3_)_2_ × 6H_2_O/17% HNO_3_)) chemical modifier were obtained from Merck (Darmstadt, Germany). An aqueous solution (deionized water) containing 0.1% (v/v) of palladium and 0.05% (v/v) of magnesium chemical modifier with 20 mg L^−1^ zirconium standard were used as HR-CS-GFMAS chemical modifier. Ten g L^−1^ of sodium acetate modifier was obtained from sodium acetate tri-hydrate (Merck, Darmstadt, Germany) in deionized water. For target analysis, mixed primary (PFAC-MXC) and ^13^C-labelled (MPFAC-C-ES and MPFAC-C-IS) PFAS standards manufactured by Wellington Laboratories (Guelph, Ontario) were purchased from Greyhound Chromatography and Allied Chemicals, UK. LC–MS grade acetonitrile and ammonium acetate used in the mobile phase for the LC–MS/MS were from Rathburn and Fisher Chemicals, respectively. LC–MS grade reagent water was also from Rathburn Chemicals.

### Sample collection

**Fig. 1:**
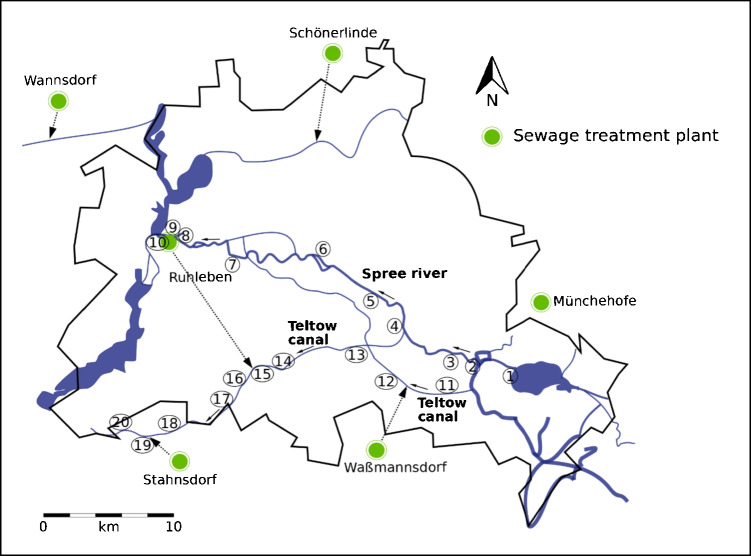
Sampling points along Spree River and Teltow Canal in Berlin, Germany. The arrows along the river and canal represent the direction of water flow. The green markings represent wastewater treatment plants (WWTPs). Arrows from the WWTPs are showing the point of discharge of effluents

About 1.5 L river water samples from Spree River, Berlin (32–40 km), and Teltow canal, Berlin (32–40 km), were collected (Spree River, 10 samples in February 2020; Teltow Canal, 10 samples in February 2020). Furthermore, a sampling campaign along the river Spree (from the east of Berlin Köpenick; to the west Spandau) and from Teltow Canal (from the east of Berlin Köpenick; to the west Dreilinden) were conducted. Coordinates of the sampling locations were tracked (Table S1, Fig. 1). Sampling was conducted as described by Metzger et al. (2019) [31] and Gehrenkemper and Simon et al. (2021) [29] to achieve the highest comparability of the results. In brief, water samples were taken near under the surface water using 2-L plastic bottle made of polypropylene (PP) mounted on a tall pole and then transported and stored in 500 mL PP bottles. Substantial losses of organofluorines could potentially be associated with sampling and storing of samples in PP tubes [36, 37], which potentially led to an underestimation of EOF and PFAS target values. Samples were collected in triplicates from each sampling point. To investigate the water phase and reduce the potential growth of microorganisms, samples were filtered using a 0.45-µm cellulose filter before storing at 4 °C.

### Sample preparation

Solid-phase extraction (SPE) was used for extraction, analyte enrichment, and sample clean-up purposes. SPE also works in eliminating unwanted inorganic fluoride. The SPE method was adapted from Metzger et al. [31] where it was validated for both ionic and neutral compounds. Briefly, Oasis HLB cartridges (Waters, 3 cc, 60 mg) were first conditioned with 3 mL methanol and then rinsed with two times with acidified deionized water (pH 2, HNO_3_). After that, the rinsing valve was closed, and the cartridges were filled with deionized acidic water of 2.5 mL (pH 2). River water samples, between 360 and 400 mL, were then loaded. After loading samples, cartridges were washed two times with 3 mL acidified deionized water (pH 2, HNO_3_). The cartridges were then dried for 30 min under vacuum and then eluted with 1 mL methanol. The eluates were evaporated to dryness using a gentle stream of nitrogen at 40 °C (SpeedVac) and reconstituted with 1 mL of water and methanol (50:50 (v/v)) mixture. Enrichment factor of about 358 was achieved, allowing for highly sensitive fluorine detection.

To demonstrate the presumption that the SPE effectively removes all inorganic fluorine, a fluoride spike (*n* = 3) was carried out with an approximately 125 µg F L^−1^ solution which was extracted the same way as the samples. Three extraction blanks that also underwent the same extraction process as the samples were included to monitor background contamination.

### Instrumental analysis

#### Target analysis

PerkinElmer Qsight LX50 UHPLC (PerkinElmer, UK) combined with a BrowLee SPP C18 column (2.7 µm, 3 × 100 mm, PerkinElmer, UK) and a BrowLee SPP guard column (2.7 µm, 3 × 5 mm, PerkinElmer, UK) was used for the separation of the analytes by LC. Five mM ammonium acetate in reagent water and 100% LC–MS grade acetonitrile (ACN) were used as mobile phase A and B for the analysis. The LC system was coupled to a PerkinElmer QSight 220 mass spectrometer (PerkinElmer, UK). Parameters for the UHPLC and optimized MS settings are given in Tables S2–S4 in the supplementary information. The MS was used in multiple reaction monitoring (MRM) mode. Two transitions (quantifier and qualifier) were monitored for the analytes except PFBA, PFPA, PFdDA, and PFOSA, where only one transition (quantifier) was monitored. Due to the large number of transitions, the transitions were monitored only around their retention times. Negative ESI mode was used for the determination of different PFAS compounds. Nitrogen was used as a collision gas with a purity of 99.9%.

#### Extractable organofluorine analysis

A ContrAA 800 high-resolution continuum source graphite furnace atomic absorption spectrometer (HR-CS-GFAAS) (Analytik Jena) with a transversely heated graphite tube atomizer was used for EOF analysis. Analysis was carried out as it was described in Metzger et al. [31], using a coated graphite furnace with integrated PIN platform (Analytical Jena); the respective temperature program is listed in Supplementary Table S5. Fluorine was detected as gallium fluoride (GaF) at a molecular absorption wavelength of 211.248 nm.

### Quality control and quality assurance

#### Target analysis

Calibration curve was prepared as a mix of PFAS analytes, in a range of 0.05 µg L^−1^ to 50 µg L^−1^ in 50% (v/v) methanol. The limit of detection (LOD) and limit of quantification (LOQ) were calculated as 3 × and 10 × the error of the *y* intercept respectively. The analytes were quantified external calibration (Fig. S7). Results between LOD and LOQ were used unchanged. In addition to the method blank, an instrumental blank of 50:50% (v/v) methanol was run at the beginning, after calibration curve and after every 20 samples. Individual PFASs detected in method blanks were subtracted from samples. Calibration curve was divided into 2, standards with high concentrations (STDH) and standards with low concentrations (STDL), and the appropriate calibration curve was used for the corresponding analyte.

To check for instrument performance, one standard was measured after every 10 samples as a quality control (QC) check. QC % was calculated by comparing the calculated concentration seen in QC checks to the theoretical concentration in µg F L^−1^.

#### EOF analysis

Since no certified reference material (CRMs) is available for PFAS analysis, an environmental chemical reference material of river water sample ION-96.4 lot 0618 (Environment and Climate Change Canada) with a certified content of 123 ± 34 µg F L^−1^ was used for checking the instrumental accuracy. Calibration curves, which bracketed the concentrations of samples, were obtained using NaF 1000 mg L^−1^ as standard stock solution.

Calibration curve in a concentration range from 5 to 100 μg F L^−1^ was prepared in a 50% (v/v) methanol. Instrumental LOD and LOQ were calculated as 3 × and 10 × the standard deviation (*n* = 10) of blanks divided by the slope of the calibration curve, respectively. For method LOD (MDL) and LOQ (MDQ), instrumental LOD and LOQ were divided by the enrichment factor (358).

## Results and discussion

### Target analysis

**Fig. 2:**
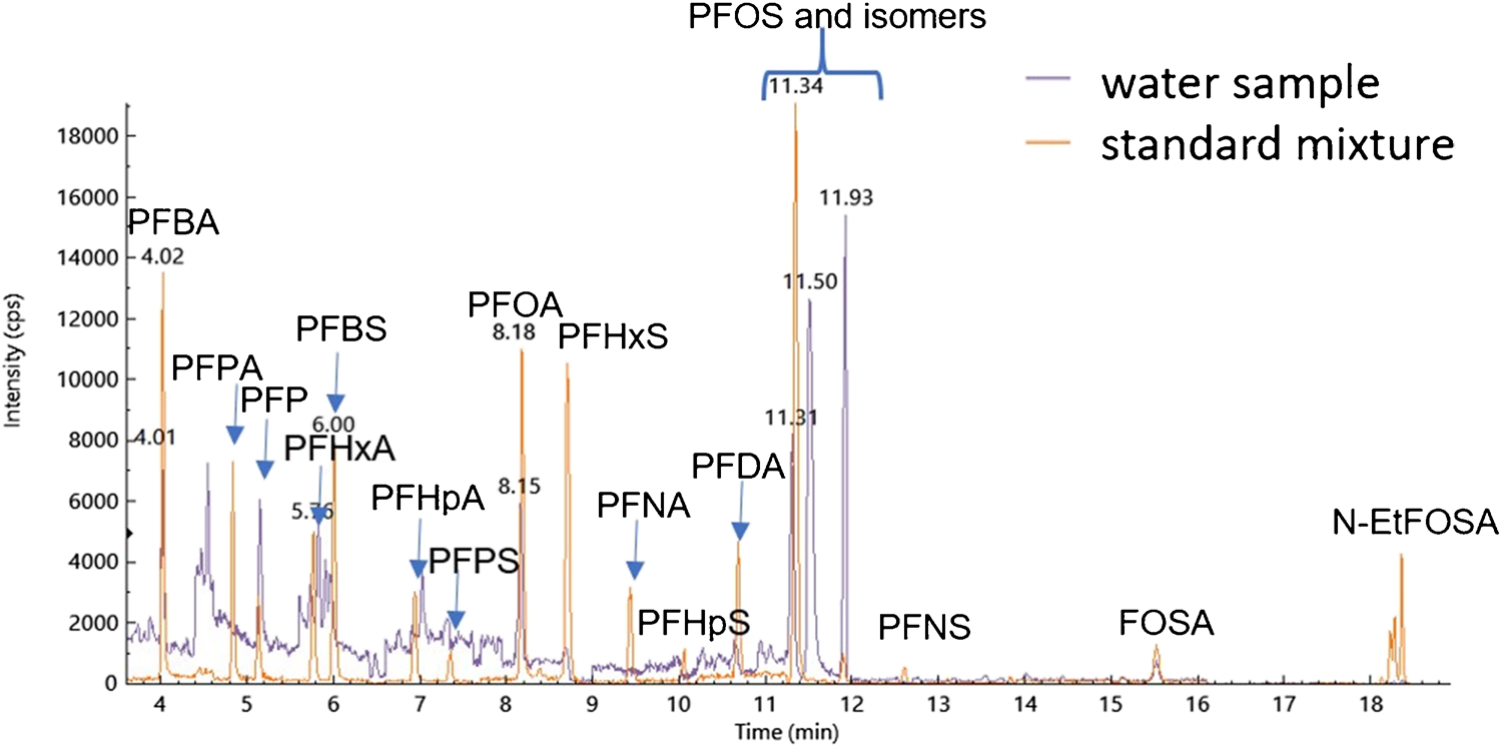
Total ion chromatogram (TIC) for a standard mix (orange) and a sample (purple) collected along River Spree, location 5 using UHPLC with C18 column (2.7 µm, 3 × 100 mm, PerkinElmer, UK) and a BrowLee SPP guard column (2.7 µm, 3 × 5 mm, PerkinElmer, UK) for separation and coupled to a PerkinElmer QSight 220 mass spectrometer (PerkinElmer, UK) operated in negative mode

Figure 2 is showing a typical total ion count (TIC) chromatogram for the 0.05 µg L^−1^ calibration mix which was used for calibration and a sample. Out of the 24 target analytes, only 10 were detected in the samples: 4 PFCAs (PFBA, PFPeA, PFHxA and PFOA), 4 PFSAs (PFPeS, PFHxS, PFHpS and PFOS), PFOSA, and PFP. Only the analytes that were detected in the samples will be analyzed here. No PFAS was found in the instrumental blanks, suggesting no leaching or carry over from the instrument. PFHxS, PFOS, PFOSA, and PFP were detected in extraction blanks. Peak areas from samples were corrected with extraction blanks for any contamination. QC checks were between 83 and 111% (Fig. S4). MDL ranged from 0.006 μg F L^−1^ (PFHxS) to 0.055 μg F L^−1^. (PFPeA) (Table S6).

PFHxA, FOSA, and PFP were found in all samples, while PFHxS and PFPeA were only found in Teltow canal. In Spree River, FOSA was the most dominant species followed by PFOA > PFHpS > PFP > PFHxA > PFPeS > PFBA, and PFOS. In Teltow canal, however, we see a profile change, possibly due to the different effluent water streams coming from the WWTPs. PFHxA is the most dominant species followed by FOSA > PFOA > PFP > PFOS > PFPeA > PFHpS > PFPeS > PFBA, and PFPeS.

**Fig. 3:**
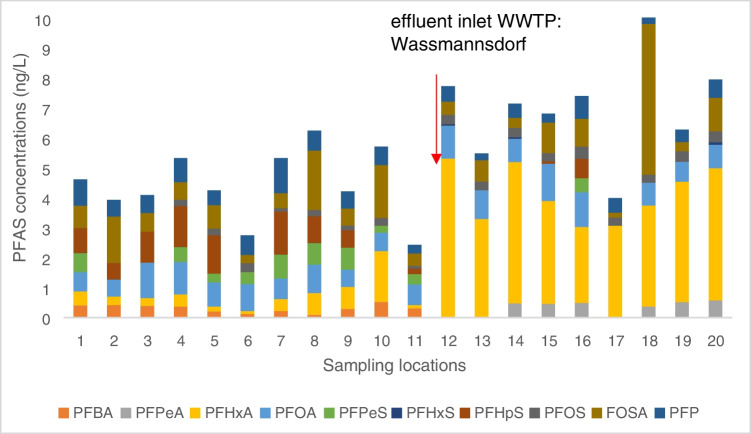
PFAS distribution in river samples (location 1–10 Spree River, location 11–20 Teltow canal)

Short-chained PFAS, such as PFHxA, were identified more often and showed higher concentrations (Table S7) (PFHxA concentration ranging from 0.1 to 5.32 ng L^−1^) than longer chained PFAS, such as PFOA and PFOS had the highest concentrations of 1.24 and 0.41 ng L^–1^, respectively. Recently, manufacturers voluntarily phased out longer chained (C < 8) PFAS due to their association with adverse health effect, such as hepatotoxicity, developmental toxicity, and hormonal effects [38–40], replacing them with smaller chained (C < 8) alternatives, leading to more frequent detection of shorter chained PFAS. Moreover, longer chain PFAS are more lipophilic; their partition coefficients (log *K*_d_) between water and sediments increase with carbon chain length [41] and therefore are more likely to be removed from the sewage water and accumulate in the sewage sludge [42]. The more water-soluble, shorter carboxylic acids (PFCA) are more likely to occur in the cleaned effluent of the sewage treatment plant. This was supported with our findings as well, since PFCAs were the highest fraction identified in the samples (Fig. S5) and no PFASs with C < 8 chain length were detected in samples. There is a clear concentration and profile change in the Teltow Canal samples between location 11 and 12 (Fig. 3) right after the effluent water income from Wassmannsdorf WWTP. From location 12 onwards, PFHxA is not only the most dominant species, but its concentration increases as well. This indicates that the effluent water coming from Wassmannsdorf WWTP is rich in PFHxA and/or its precursors. It is known [43–45] that perfluorinated phosphate esters (PAPs) and fluorotelomers (FTOHs) which have not been measured with the targeted analysis could degrade to PFHxA and other perfluorinated carboxylic acids, increasing their concentrations. We also found an increase in PFHxA along the Spree River from location 6 onwards; however, there is no indication of any water discharge at that point.

### Total EOF and mass balance analyses

Instrumental LOD is calculated to be 0.43 μg F L^−1^, which is lower than what was reported previously [29, 31], using the same method. MDL and MDQ were calculated to be of 1.2 ng F L^−1^ and 4 ng F L^−1^ in the sampled water. We found that more than 99.9% of the originally spiked fluoride was removed during SPE process (Table S8), meaning that results obtained from EOF analysis represent the organically bound fluorine fraction. Analysis of CRM showed good agreement with the certified value (103 ± 6.9% recovery). All measured river water samples contained quantifiable concentrations of EOF (Table S9a) ranging from 40 ng F L^−1^ (location 11) to 580 ng F L^−1^ (location 14). Previous reports on water samples showed an EOF of 93 ng F L^−1^ and 562 ng F L^−1^ in non-contaminated and contaminated seawater, respectively [25] 42–550 μg F L^−1^ [31] and 5–300 ng F L^−1^ [29] in different rivers in Germany, which agrees with our findings.

#### Spree River

**Fig. 4:**
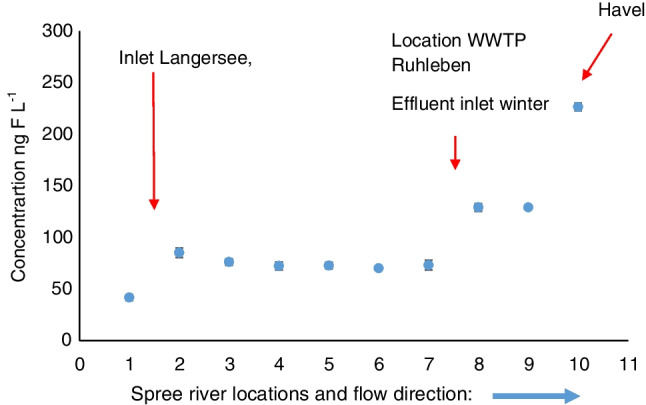
Concentrations of EOF in Spree River samples (Berlin); the error bars indicate 1 SD of the EOF results (*n* = 3)

EOF concentrations in Spree River ranged from 50 ng F L^−1^ (location 1) to 229 ng F L^−1^ (location 10). Locations 8 to 10 had more than 2 times higher EOF than location 1–7 (Fig. 4). This was expected due to the proximity to the Ruhleben sewage plant which is a potential source of PFASs. Additional discharge of Ruhleben sewage plant into the Spree in case of rainy weather. We also expect an increase in EOF concentration close to the Muenchehofe sewage treatment plant; however, there is no evidence that the sewage plant releases effluent water into the canal. (The Muenchehofe sewage treatment plant releases effluent water into the “Neuenhagener Mühlenfließ” outside of Berlin.)

#### Teltow Canal

**Fig. 5:**
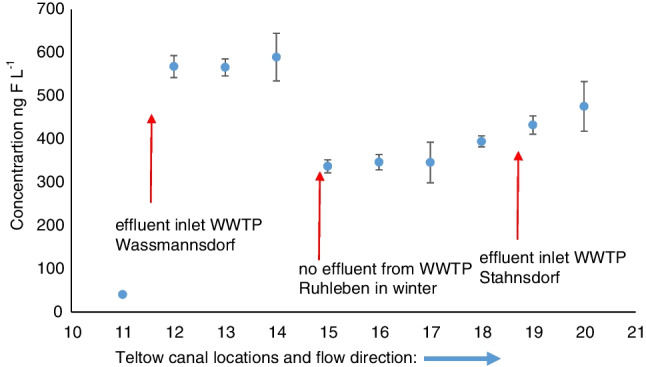
Concentrations of EOF in Teltow Canal samples (Berlin); the error bars indicate the standard deviation of the EOF results (*n* = 3)

EOF concentrations in Teltow canal ranged from 40 ng F L^−1^ (location 11) to 580 ng F L^−1^ (location 14) (Fig. 5). Water sampled from sampling point 11 had the lowest EOF concentration probably due its location before the Wassmannsdorf sewage treatment plant. Locations from 12 to 14 had the highest EOF concentrations, all around 580 ng F L^−1^ which is more than 14 × higher than location 11. This could be the result of a discharge point from the Wassmannsdorf wastewater treatment plant (WWTP) which is located between 11 and 12. The increase seen in the EOF concentrations indicates that effluent water from Wassmannsdorf WWTP contains significant amount of PFAS. There was a decrease in EOF concentration to around 300 ng F L^−1^ in locations 15 to 17. No effluent from WWTP Ruhleben was discharged here in the winter when sampling took place. The effluent from the WWTP Ruhleben goes directly at the location into the river Spree. An explanation for the observed decrease in EOF could be that there is a tributary between sampling points 14 and 15 which dilutes the EOF concentration in the canal. Another explanation could be that a discharge here could add suspended solids which would be a sink of PFAS in the water column. PFAS and other less soluble organofluorines would adsorb on the surface of the particles and then sedimented in the low flow of the canal. From location 17 onwards, the EOF concentrations increased steadily in the direction of flow. Stahnsdorf WWTP has an effluent water stream between locations 18 and 19, which could explain the increase in EOF concentrations.

Generally, higher EOF concentrations (by a factor of 2 and 3) were found in water samples from Teltow canal compared to those from Spree River (Table S9a, Figure). Teltow canal flows slower than the Spree River; thus, the water turnover is smaller, leading to higher concentrations of individual PFASs in the canal. Another explanation could be that simply more PFAS was released into the Teltow canal than into Spree River. To assess this possibility, effluent water from the WWTPs need to be sampled.

**Fig. 6:**
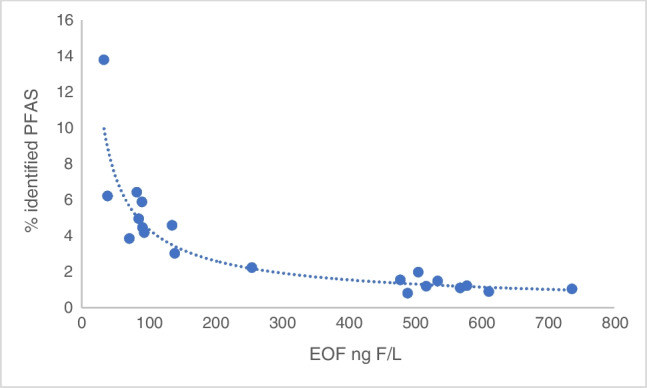
Correlation of identified PFAS (target analysis) versus EOF

**Fig. 7:**
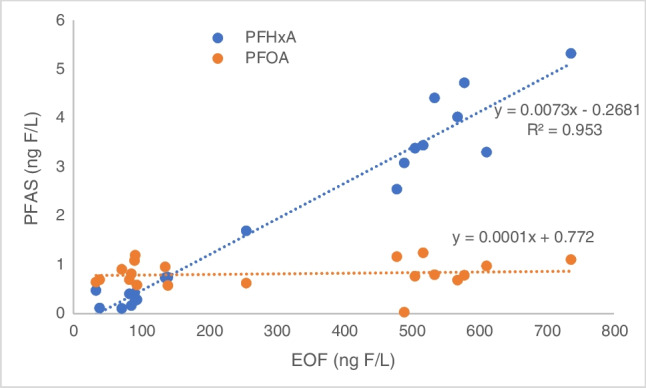
Correlation of selected PFAS (PFHxA and PFOA) versus EOF

Individual PFAS concentrations were converted into ng F L^−1^ and compared to the EOF. ∑identified PFAS accounted for 0.81 to 13.8% of the total EOF (Table 1). This is in agreement with previous findings where the majority of EOF (up to 99.8%) remained unidentified by target analysis from water samples [46]. The proportion of identified PFAS from the targeted analysis decreases exponentially with the EOF (Fig. 6). This suggests that the increase of EOF downstream from the sewage inlets is mainly caused by PFAS or other organofluorinated pharmaceuticals not targeted in the analysis. However, the concentration increases of PFHxA along the Teltow canal in particular shows a positive correlation with EOF concentrations, whereas PFOA does stay constant (Fig. 7). This suggests that EOF (across a series of samples) could be described with only monitoring PFHxA concentrations. However, PFHxA is only a small fraction of EOF (< 5%); therefore, it cannot be used as a quantifiable unit for EOF. Whether the increase is a source of PFHxA precursors not monitored and unknown or the correlation is entirely a coincidence. Hence, more studies are required with different sampling points to properly assess the correlation between PFHxA and EOF and the use of suspected screening or non-targeted analysis using accurate mass spectrometry. Notably, EOF can contain not only PFAS but fluorinated pharmaceuticals as well, which are not at all monitored with target PFAS methods and might not have any available reference standards for analysis. Hence, a non-target approach and more focus on fluorinated pharmaceuticals are also needed to identify more of the unknown EOF content.

**Table 1:** EOF mass balance in river water samples

Location	C_F_ ∑PFAS_ (ng F L^−1^)	EOF (ng F L^−1^)	C_F_ ∑PFAS_/EOF (%)
1	4.62	33.6	13.8
2	3.94	94.2	4.2
3	4.09	91.6	4.5
4	5.34	90.7	5.9
5	4.25	85.9	5.0
6	2.76	71.8	3.9
7	5.33	83.0	6.4
8	6.25	136	4.6
9	4.23	140	3.0
10	5.71	256	2.2
11	2.43	39.0	6.2
12	7.74	737	1.1
13	5.50	612	0.9
14	7.14	579	1.2
15	6.81	518	1.2
16	7.43	479	1.6
17	3.98	490	0.8
18	10.0	506	2.0
19	6.28	569	1.1
20	7.96	535	1.5

It should be noted that since 2005, waste waters from industry and domestic use in Berlin should be discharged to central sewage treatment plants. Therefore, industrial waste waters are directed to the centralized WWTPs before they are discharged into the environment. It is possible to set up a decentralized, state-of-the-art waste water treatment plant in a few justified exceptional cases. The authors are not aware of such initiatives with respect to the investigated areas in Berlin. Therefore, the investigated parameters EOF and PFAS most likely reflect both industrial as well as domestic entry sources of PFAS and other organofluorines into surface water bodies in Berlin explainable by discharges of the centralized WWTPs.

## Conclusion

In this study, we focused on identifying the overall organofluorine pollution via sum parameters, using HR-CS-GFMAS and target analysis for PFAS. We showed that target analysis accounts for a maximum of 13.8% of EOF. EOF concentration revealed potential “hotspots” along the Spree River and Teltow canal with the highest concentrations of EOF being found along the Teltow Canal, after the suspected source of contamination. EOF concentrations increased along the river flow by one order of magnitude, which has been recorded in the canal system in Berlin, indicating that cleaned sewage may contain significant amounts of EOF. Due to the non-disclosure and little information on the newly produced replacement PFASs and fluorinated pharmaceuticals, researchers face a difficulty identifying novel compounds in the environment. As a result, their environmental distribution remains unknown. The high percentage of unidentified PFASs shows the importance for the development of new sensitive analytical methods to determine a sum parameter for PFAS and other organofluorine compounds.

## Supplementary Information

Below is the link to the electronic supplementary material.

Supplementary file1 (DOCX 1585 KB)
