# Supplementary material for: EOF and target PFAS analysis in surface waters affected by sewage treatment effluents in Berlin, Germany
Source: Anal Bioanal Chem. 2023 Jan 12;415(6):1195–204. doi: 10.1007/s00216-022-04500-x (PMC9899726; doi:10.1007/s00216-022-04500-x)
Supplement: Supplementary file 1 — Supplementary file1 (DOCX 1585 KB) [file 216_2022_4500_MOESM1_ESM.docx]

# Supplementary information

# EOF and target-PFAS analysis in surface waters affected by sewage treatment effluents in Berlin, Germany

**Table S1:** Coordinates for sampling points

| Sample location | Coordinates | |
| --- | --- | --- |
| 1 | N 52°26.678' | E013°37.376 |
| 2 | N 52°26.900' | E 013°34.210' |
| 3 | N 52° 27.186' | E 013°33.324' |
| 4 | N 52°28.331' | E 013°29.706' |
| 5 | N 52°28.331' | E 013°28.222' |
| 6 | N 52°31.199' | E 013°24.090' |
| 7 | N 52°31.291' | E 013°18.162' |
| 8 | N 52°32.024' | E 013°14.004' |
| 9 | N 52°32.085' | E 013°13.668' |
| 10 | N 52°32.138' | E 013°12.486' |
| 11 | N 52°31.582' | E 013°13.298' |
| 12 | N 52°25.672' | E 013°30.433' |
| 13 | N 52°27.557' | E 013°26.887' |
| 14 | N 52°26.586' | E 013°21.248' |
| 15 | N 52°26.697' | E 013°19.927' |
| 16 | N 52°26.234' | E 013°19.174' |
| 17 | N 52°26.236' | E 013°19.176' |
| 18 | N 52°25.165' | E 013°18.155' |
| 19 | N 52°27.723' | E 013°12.469' |
| 20 | N 52°23.980' | E 013°10.776' |

**Table S2:** Instrumental Parameters for UHPLC

| **Analytical Column** | Brownlee, SPP C18, 100 × 3.0 mm, 2.7 μm |
| --- | --- |
| **Guard Column** | Brownlee, SPP C18, 5 × 3.0 mm, 2.7 μm |
| **Mobile phase A** | 5 mM ammonium acetate in Milli-Q water |
| **Mobile phase B** | LCMS grade Acetonitrile |
| **Column Oven Temperature** | 30 °C |
| **Flow rate** | 0.3 mL min^-1^ |
| **Injection volume** | 10 μL |

**Table S3:** HPLC gradient for separation of analytes

| Time | % A | % B |
| --- | --- | --- |
| 0.0 | 90 | 10 |
| 1.0 | 90 | 10 |
| 3.0 | 70 | 30 |
| 15.0 | 35 | 65 |
| 17.1 | 2 | 98 |
| 20.1 | 2 | 98 |
| 20.3 | 90 | 10 |
| 22.2 | 90 | 10 |

**Table S4:** Monitored mass-to-charge (*m/z*) ratios, optimised instrument parameters along with retention times (RT) in minutes

|  | **Standards** | **Acronym** | **Q1** | **Q2** | **RT** | **CE** | **EV** | **CCL2** |
| --- | --- | --- | --- | --- | --- | --- | --- | --- |
|  | **Perfluoro Carboxylic Acids (PFCAs)** | | | | | | | |
| **Acids** | perfluorobutanoic acid | PFBA | 213 | 169 | 4.1 | 11 | -1 | 36 |
|  |  |  | 213 | 19.4 | 4.1 | 144 | -10 | 64 |
|  | perfluoropentanoic acid | PFPeA | 263 | 219 | 4.9 | 10 | 0 | 44 |
|  | perfluorohexanoic acid | PFHxA | 313 | 269 | 5.9 | 13 | 0 | 48 |
|  |  |  | 313 | 119 | 5.9 | 34 | 0 | 76 |
|  | perfluoroheptanoic acid | PFHpA | 363 | 319 | 7.1 | 13 | -8 | 84 |
|  |  |  | 363 | 169 | 7.1 | 25 | -12 | 92 |
|  | perfluorooctanoic acid | PFOA | 413 | 369 | 8.2 | 14 | -14 | 68 |
|  |  |  | 413 | 169 | 8.2 | 27 | 0 | 72 |
|  | perfluorononanoic acid | PFNA | 463 | 419 | 9.5 | 15 | -9 | 100 |
|  |  |  | 463 | 219 | 9.5 | 21 | -12 | 92 |
|  | perfluorodecanoic acid | PFDA | 513 | 469 | 10.7 | 15 | 0 | 88 |
|  |  |  | 513 | 219 | 10.7 | 24 | -16 | 124 |
|  | perfluoroundecanoic acid | PFUdA | 563 | 519 | 12.0 | 16 | -6 | 100 |
|  |  |  | 563 | 169 | 12.0 | 32 | -13 | 92 |
|  | perfluorododecanoic acid | PFDoA | 613 | 569 | 13.2 | 15 | -2 | 96 |
|  | perfluorotridecanoic acid | PFTrDA | 663 | 619 | 14.4 | 18 | -7 | 136 |
|  |  |  | 663 | 169 | 14.4 | 3 | -10 | 116 |
|  | perfluorotetradecanoic acid | PFTeDA | 713 | 669 | 15.6 | 21 | -20 | 156 |
|  |  |  | 713 | 169 | 15.6 | 48 | -23 | 128 |
|  | perfluorohexadecanoic acid | PFHxDA | 813 | 769 | 17.3 | 14 | -36 | 108 |
|  |  |  | 813 | 169 | 17.3 | 43 | -5 | 200 |
|  | perfluorooctadecanoic acid | PFODA | 913 | 869 | 17.8 | 17 | -36 | 108 |
|  |  |  | 913 | 169 | 17.8 | 39 | -16 | 156 |
|  | **Perfluoro Sulfonic Acids (PFSAs)** | | | | | | | |
|  | perfluoro-1- butanesulfonic acid | PFBS | 299 | 80 | 6.1 | 75 | -22 | 104 |
|  |  |  | 299 | 99 | 6.1 | 48 | -1 | 76 |
|  | perfluoro-1-pentanesulfonic acid | PFPeS | 349 | 80 | 7.4 | 61 | -41 | 92 |
|  |  |  | 349 | 99 | 7.4 | 47 | -34 | 88 |
|  | perfluoro-1-hexanesulfonic acid | PFHxS | 399 | 80 | 8.8 | 50 | -52 | 76 |
|  |  |  | 399 | 99 | 8.8 | 45 | -35 | 152 |
|  | perfluoro-1-heptanesulfonic acid | PFHpS | 449 | 80 | 10.1 | 88 | -40 | 124 |
|  |  |  | 449 | 99 | 10.1 | 49 | 0 | 108 |
|  |  |  | 499 | 80 | 11.4 | 99 | -54 | 156 |
|  |  |  | 499 | 99 | 11.4 | 57 | -63 | 112 |
|  | perfluoro-1-nonanesulfonic acid | PFNS | 549 | 80 | 12.7 | 107 | -62 | 180 |
|  |  |  | 549 | 99 | 12.7 | 63 | -52 | 132 |
|  | perfluoro-1-decanesulfonic acid | PFDS | 599 | 80 | 13.9 | 125 | -63 | 172 |
|  |  |  | 599 | 99 | 13.9 | 75 | -50 | 212 |
|  | perfluoro-1-dodecanesulfonic acid | PFDoS | 699 | 80 | 16.3 | 125 | -63 | 172 |
|  |  |  | 699 | 99 | 16.3 | 75 | -50 | 212 |
|  |  |  |  |  |  |  |  |  |
|  | **Sulfonamides and phenol** | | | | | | | |
| **Other** | perfluorooctane sulfonamide | PFOSA | 498 | 78 | 15.5 | 53 | -36 | 112 |
|  | N-ethyl perfluorooctyl sulphonamide | N-EtFOSA | 526 | 169 | 18.4 | 34 | -6 | 128 |
|  |  |  | 526 | 219 | 18.4 | 31 | -7 | 140 |
|  | Pentafluorophenol | PFP | 183 | 117 | 5.1 | 26 | -26 | 64 |

**Table S5:** Temperature program for EOF determination via GaF in a Zr-coated graphite furnace platform

| Step | T / ^o^C | Ramp / ^o^ C s^-1^ | Hold / s |
| --- | --- | --- | --- |
| Drying | 80 | 5 | 25 |
| Drying | 90 | 5 | 30 |
| Drying | 110 | 5 | 20 |
| Pyrolysis | 500 | 500 | 10 |
| Gas adaption | 500 | 0 | 5 |
| Atomization | 1550 | 1500 | 6 |
| Cleaning | 2450 | 500 | 5 |

**Table S6a:** Limits of detection and limits of quantification for individual target PFASs of the instrument (μg F L^-1^)

| **Parameter** | **PFBA** | **PFPeA** | **PFHxA** | **PFOA** | **PFHxS** | **PFHpS** | **PFOS** |
| --- | --- | --- | --- | --- | --- | --- | --- |
| LOD | 0.020 | 0.055 | 0.024 | 0.036 | 0.006 | 0.006 | 0.023 |
| LOQ | 0.062 | 0.166 | 0.074 | 0.108 | 0.019 | 0.019 | 0.070 |

**Table S6b:** Limits of detection and limits of quantification for individual target PFASs in the water samples with the average enrichment factor of 265 (ng F L^-1^).In some cases the enrichment factor was significant larger which result into lower LOD and LOQs.

| **Parameter** | **PFBA** | **PFPeA** | **PFHxA** | **PFOA** | **PFHxS** | **PFHpS** | **PFOS** |
| --- | --- | --- | --- | --- | --- | --- | --- |
| LOD | 0,078 | 0.21 | 0.093 | 0.14 | 0.024 | 0.024 | 0.088 |
| LOQ | 0.23 | 0.63 | 0.28 | 0.41 | 0.07 | 0.07 | 0.26 |

**Table S7:** List of target PFASs detected from each of the sampling locations, their concentrations (ng F L^-1^)

| **Sampling Point** | **PFBA** | **PFPeA** | **PFHxA** | **PFOA** | **PFPeS** | **PFHxS** | **PFHpS** | **PFOS** | **FOSA** | **PFP** |
| --- | --- | --- | --- | --- | --- | --- | --- | --- | --- | --- |
| 1 | 0.41 | <LOD | 0.47 | 0.64 | 0.64 | <LOD | 0.84 | <LOD | 0.74 | 0.88 |
| 2 | 0.42 | <LOD | 0.28 | 0.58 | <LOD | <LOD | 0.55 | <LOD | 1.55 | 0.55 |
| 3 | 0.39 | <LOD | 0.26 | 1.19 | <LOD | <LOD | 1.03 | <LOD | 0.63 | 0.59 |
| 4 | 0.37 | <LOD | 0.41 | 1.08 | 0.50 | <LOD | 1.37 | 0.21 | 0.59 | 0.80 |
| 5 | 0.21 | <LOD | 0.16 | 0.81 | 0.29 | <LOD | 1.29 | 0.22 | 0.78 | 0.49 |
| 6 | 0.12 | <LOD | 0.10 | 0.90 | 0.41 | <LOD | <LOD | 0.29 | 0.28 | 0.65 |
| 7 | 0.22 | <LOD | 0.40 | 0.69 | 0.80 | <LOD | 1.44 | 0.11 | 0.50 | 1.17 |
| 8 | 0.09 | <LOD | 0.73 | 0.95 | 0.73 | <LOD | 0.89 | 0.21 | 1.99 | 0.66 |
| 9 | 0.29 | <LOD | 0.74 | 0.57 | 0.74 | <LOD | 0.58 | 0.16 | 0.57 | 0.57 |
| 10 | 0.53 | <LOD | 1.69 | 0.62 | 0.24 | <LOD | <LOD | 0.25 | 1.77 | 0.61 |
| 11 | 0.31 | <LOD | 0.11 | 0.69 | 0.34 | <LOD | 0.19 | 0.09 | 0.42 | 0.28 |
| 12 | <LOD | <LOD | 5.32 | 1.10 | <LOD | 0.05 | <LOD | 0.31 | 0.44 | 0.51 |
| 13 | <LOD | <LOD | 3.30 | 0.97 | <LOD | <LOD | <LOD | 0.28 | 0.71 | 0.23 |
| 14 | <LOD | 0.48 | 4.72 | 0.78 | <LOD | 0.05 | <LOD | 0.32 | 0.33 | 0.47 |
| 15 | <LOD | 0.46 | 3.44 | 1.24 | <LOD | <LOD | 0.09 | 0.27 | 1.02 | 0.29 |
| 16 | <LOD | 0.49 | 2.54 | 1.16 | 0.47 | 0.07 | 0.65 | 0.41 | 0.93 | 0.76 |
| 17 | <LOD | <LOD | 3.08 | <LOD | <LOD | 0.07 | <LOD | 0.26 | 0.16 | 0.48 |
| 18 | <LOD | 0.37 | 3.38 | 0.76 | <LOD | <LOD | <LOD | 0.27 | 5.04 | 0.21 |
| 19 | <LOD | 0.52 | 4.02 | 0.68 | <LOD | <LOD | <LOD | 0.34 | 0.31 | 0.41 |
| 20 | <LOD | 0.58 | 4.41 | 0.79 | <LOD | 0.08 | <LOD | 0.37 | 1.12 | 0.61 |

**Table S8:** Measurement of the method blank and 125 μg L^-1^ fluoride solution after extraction with SPE

| **Sample** | **EOF concentration**  **(ng F L^-1^)** | **SD (ng F L^-1^)** | **RSD (%)** |
| --- | --- | --- | --- |
| Method blank | 2.2 | 0.13 | 5.8 |
| Blank spiked with 125 μg F L^-1^ | 1.2 | 0.23 | 19 |

**Table S9a**: Mean concentrations for EOF (ng L^-1^) ± 1 standard deviation for the different sampling locations (n = 3)

| Sample location | EOF concentrations (ng L^-1^) |
| --- | --- |
| 1 | 41.8 ± 2.8 |
| 2 | 85.2 ± 4.6 |
| 3 | 76.2 ± 3.2 |
| 4 | 72.3 ± 3.8 |
| 5 | 72.6 ± 3.2 |
| 6 | 70.1 ± 1.5 |
| 7 | 73.1 ± 4.8 |
| 8 | 129 ± 3.8 |
| 9 | 129 ± 1.3 |
| 10 | 226 ± 3.8 |
| 11 | 40.3 ± 2.6 |
| 12 | 567 ± 26 |
| 13 | 566 ± 20 |
| 14 | 589 ± 55 |
| 15 | 337 ± 15 |
| 16 | 346 ± 18 |
| 17 | 345 ± 47 |
| 18 | 394 ± 13 |
| 19 | 401 ± 18 |
| 20 | 475 ± 58 |

**Table S9b:** Comparison of EOF before effluent inlet and downstream. All concentrations in ng F L^-1^.

| Sampling location | Mean EOF before effluent | Mean EOF after effluent | Increase of EOF | Factor increase | Potential effluent source |
| --- | --- | --- | --- | --- | --- |
| Spree 1 to 2 | 41.8 | 85.2 | 43.4 | 2.04 | WWTP Münchehofe |
| Spree 2-7 to 8-10 | 74.9 | 161 | 86.1 | 2.15 | WWTP Ruhleben / River Havel |
| Teltow 11 to 12-14 | 40.3 | 574 | 534 | 14.2 | WWTP Waßmannsdorf |
| Teltow 12-14 to 15-17 | 574 | 343 | -231 | 0.60 | WWTP Ruhleben |
| Teltow 15-17 to 18-20 | 343 | 434 | 91 | 1.27 | WWTP Stahnsdorf |


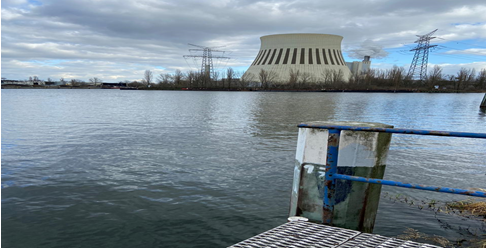


**Figure S1:** Sample location 8 from Spree River


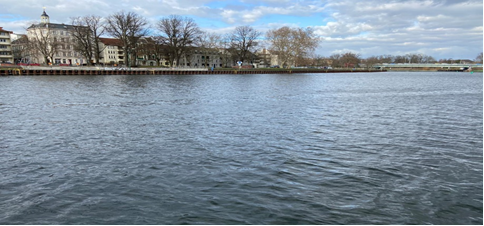


**Figure S2:** Sample location 10 from Spree river


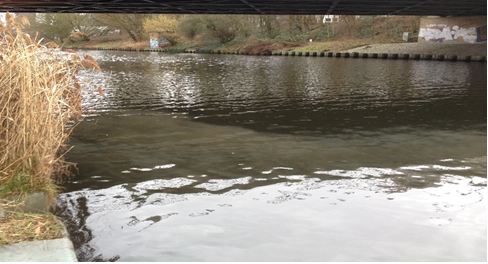


**Figure S3:** Sample location 17 from Teltow canal

**Figure S4a:** QC% for PFAS analytes using LC-MS/MS. Error bars represent relative standard deviation (%) for n = 3.

**Figure S4b:** Visual illustration of instrumental accuracy of QC standards from the PFAS analysis of ~2.5 µg F L^-1^ standard over three days of measurements demonstrating the reproducibility.

**Figure S5:** Contribution profile (%) for each class of PFAS (PFCA: Perfluorinated carboxylic acids and PFSA: perfluorinated sulfonamids).


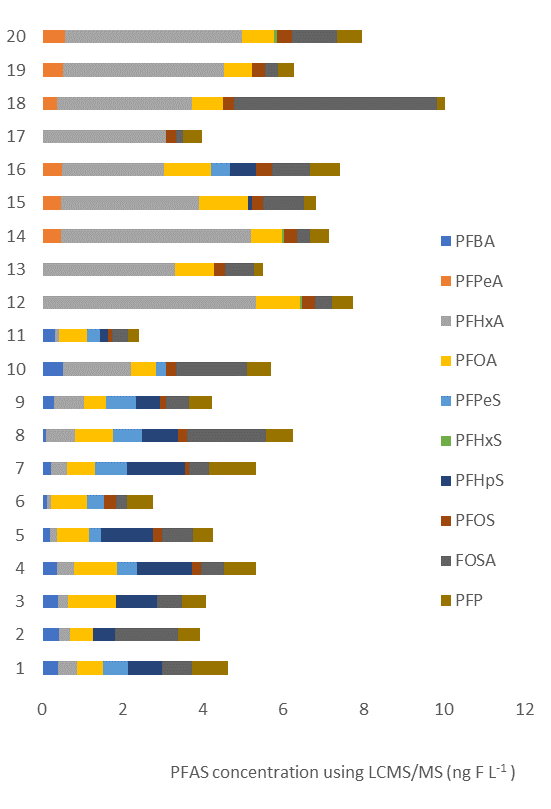

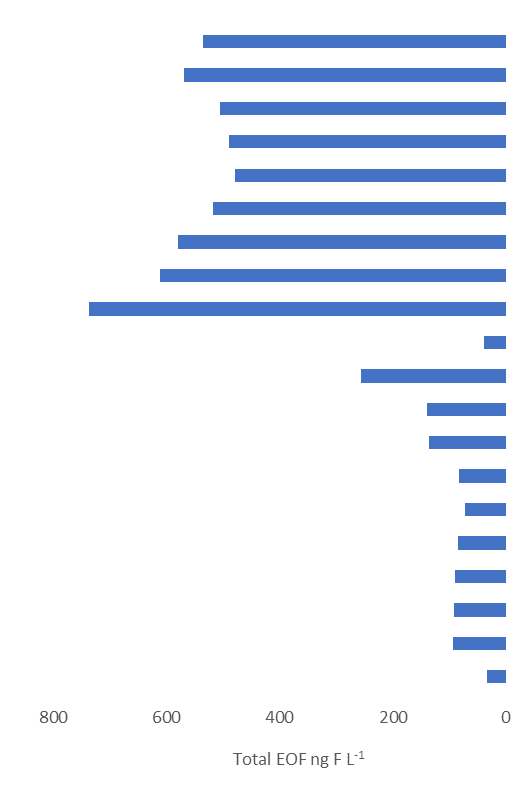


**B**

**A**

**Figure S6:** (A) Total EOF (ng F L^-1^) determined using HR-CS-GFMAS and (B) PFAS concentrations (ng F L^-1^) determined using HPLC-MS/MS operated in negative mode


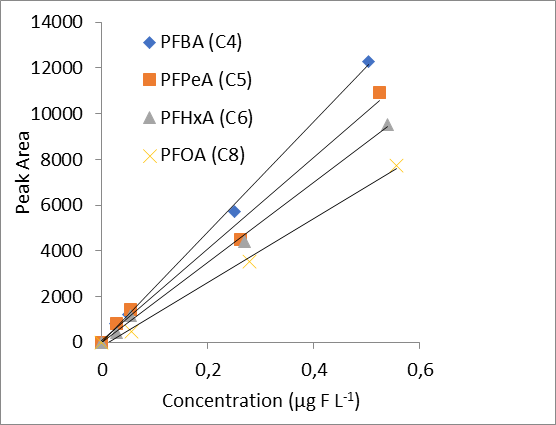

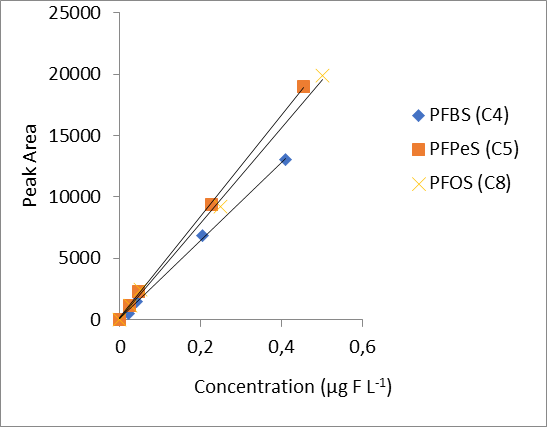


**Figure S7:** Calibration curves for the individual PFASs with HPLC-MS/MS.
